# Mr. Besant on Drunkenness
*"The Demoniac." By Walter Besant. Arrowsmith's Christmas Annual, 1890. (Arrowsmith, Bristol. One Shiling.)


**Published:** 1890-11-22

**Authors:** 


### Turning Over New Leaves.

### Mr. Besant on Drunkenness.*

Formerly Christmas was a merry time, a time of beef and pudding, of wine and wassail, of re-union of friends and forgiveness of enemies. Then literature reflected the spirit of the age, and our Christmas books were bright with peace and goodwill, with happy coincidences, with joyous accidents such as do happen occasionally in real life, though not so often there as in the books. But now! Are we growing so sad and scientific in the world's old age that even at Christmas we cannot make merry? And is Mr. Besant, who has so often told us those bright impossible stories that after all make us more kindly and helpfully disposed to each other—is Mr. Besant also among the dark prophets of realism? "The Demoniac," his Christmas annual for this year, is one of the saddest stories ever penned, the story of a man, young, rich, and talented, who finds he is the victim of a hereditary craving for strong drink. The first attack comes on suddenly, irresistibly, when he is about twenty-one, and the paroxysms recur with absolute precision every two months for five or six years, when, as a last rebellion against his shame, he drowns himself. This is, in outline, the story Mr. Besant tells; its main incidents are George Atheling's efforts to overcome his vice, his precautions, which, when the moment of temptation comes, no one is more anxious to evade than he, his casting away of home, friends, and fortune in the attempt to fly from himself, his discovery, and his death at the moment when redemption seems most possible.

The book is a nightmare, born of crude science imperfectly amalgamated with fiction. The ways of heredity are not so plain, so sharp and clear-cut as Mr. Besant believes. Drunkenness is a disease, frequently inherited—and, as in George Atheling's case, often characterised by atavism—but its onsets are not such as those described. They begin more insidiously and develop more gradually, nor is their periodicity as regular as Mr. Besant seems to think. A man who begins with an attack of dipsomania every two months would, in less than five years, have grown to be an open drunkard. He would not have had strength to keep from touching the fortune that gave him such large possibilities of indulgence; he would have ceased to care whether or not his wife knew of his sin; he would have been on the high road to indifference to the world's esteem. Inebriety, like other diseases, becomes chronic as time goes on; the attacks become more frequent and the resistance less. Atheling would not have gone away to the retirement in which he yielded to his vice with secret shame but with secret longing, and his whole nature would have sunk far lower than it does. For though a physician supposed to be dowered with preternatural insight—though he makes one most egregious blunder—tells Atheling that his nature will deteriorate under the influence of drink, the deterioration does not come off. He is a better man the day he dies, and even before that, before the struggle for retrieval has begun, than he was when he took his name off the college books because he had found he was a drunkard. Drunkards are spasmodically affectionate and industrious, but they do not, like Atheling, make good husbands nor good sub-editors. Mr. Besant has grasped clearly and presented vividly the great and pitiable fact of hereditary inebriety; but he has not carefully enough studied its symptoms nor marked its development.

On one other point we join issue with Mr. Besant—the blunder made by the before-mentioned physician. This man of genius and wide experience sends two young doctors, strong, healthy, intelligent fellows, on a yachting tour round the world with Atheling, giving them instructions to watch him continually, and prevent him yielding to his drunken fits. And he actually succeeds in finding for the post two such born innocents—to use a kindly word—that when after their charge has once locked his cabin door during the attack, and they, watching outside, see that no liquor-goes in, yet find him drunk, with the cabin smelling of whiskey when the door is opened, they actually go on with the same futile performance every other month for two whole years. It does not strike either of them to insist on watching inside the cabin, and fighting for the right to do so, although their muscular capacity is one of their strong points. They return home, having evolved a theory of the "unconscious simulation of alcoholic symptoms"—including the simulated, but quite perceptible, alcoholic smell—wherewith they purpose to astound the world. Now where, in all the ranks of medical studentdom, did the physician find two such babes? Mr. Besant says it was at St. George's. This must be a libel. If all the medical schools in the kingdom were searched, one such strabismal idiot might be found—though hardly in the form of a sane strong young giant like the men described—but it is not possible that there could be two.

The fact is, Mr. Besant, in striving to prove his point—the cunning and determination of the drunkard—has too palpably made everything and everyone play into Atheling's hands. We deny the young doctors absolutely; they are more mythical than their own theory; and we can hardly accept the wife who so implicitly accepts the story of business in Boston demanding attention every two months, and never inquires its nature. We know one or two women, therefore we are sceptical. Nevertheless, we are glad of Nettie and her family, and all the inhabitants of the pleasant though impecunious suburb here called Clerkland. We like to hear about their lives—the "managing" capacity of the women, and the "genteel," albeit somewhat narrow and priggish respectability of the men. These clerks and their wives are Mr. Besant's own; he knows them better than they know themselves, and touches on their merits and their faults with a pencil true to life, yet dipped in the colours of romance. We would rather, even at the risk of monotony, meet him in their company than in the gloomy regions which this year he has elected to visit, and where he is wholly unromantic, yet not, by way of compensation, true.

* "The Demoniac." By Walter Besant. Arrowsmith's Christmas Annual, 1890. (Arrowsmith, Bristol. One Shilling.)

